# Predictions of energy efficient Berger-Levy model neurons with constraints

**DOI:** 10.1186/1471-2202-14-S1-P349

**Published:** 2013-07-08

**Authors:** Siavash Ghavami, Farshad Lahouti, Lars Schwabe

**Affiliations:** 1 School of Electrical and Computer Engineering, University of Tehran, Tehran, 14395-515, Iran; 2 Faculty of Computer Science and Electrical Engineering, Universität Rostock, 18059, Germany

## 

Information theory has been extensively applied to neuroscience problems. The mutual information between input and output has been postulated as an objective, which neuronal systems may optimize. However, only recently the energy efficiency has been addressed within an information-theoretic framework [1]. Here, the key idea is to consider capacity per unit cost (measured in bits per joule, bpj) as the objective. We are interested in how biologically plausible constraints affect predictions made by this new theory for bpj-maximizing model neurons.

More specifically, in our contribution, in line with [1] and [2], a neuron is modeled as a memory-less constant communication channel with a Gamma conditional probability distribution function (PDF) [1]. In this setting, the channel input and output are the excitatory postsynaptic potential intensity,  λ, and the inter spike interval (ISI),  t, with PDFs $f_{\Lambda }\left(\lambda \right)$ and $f_{T}\left(t\right)$, respectively. We then formulate two new constraints: First, we impose a lower bound $t_{\text{min}}$ on the duration  tof ISIs. The rational for this is to account for a maximal firing rate. Second, we consider a peak energy expenditure constraint per ISI as compared to only bounding the expected energy expenditure. This translates into an upper bound $t_{\text{max}}$ on the ISI duration. We then derive the $f_{T}\left(t\right)$ (corresponding to valid $f_{\Lambda }\left(\lambda \right)$) of a bpj-maximizing neuron for the original unconstrained setting from [1] and in the presence of the above two constraints for different expected ISIs. (Details omitted here for brevity.) Figure 1 shows three $f_{T}\left(t\right)$s obtained in the unconstrained (dashed curves) and constrained settings (solid curves) for $t_{\text{min}}=1$and $t_{\text{max}}=5$. While the constrained and unconstrained solutions have the same mean, the shape of their $f_{T}\left(t\right)$ differ. For comparison with experimental data, we computed the coefficient of variation (CV) as a function of the mean ISI as an "observable" (Figure 2), which is easier to measure experimentally than the full distribution $f_{T}\left(t\right)$. Interestingly, the CV is predicted i) to be lower in the constrained setting, and ii) to *increase *and then decrease with the mean ISI while it only *decreases *in the unconstrained setting. Thus, we demonstrated that constraints can affect predictions based on bpj-maximization, and should be explicitly taken into account. Ongoing work makes these predictions more quantitative via simulating biophysically realistic model neurons.

**Figure 1:**
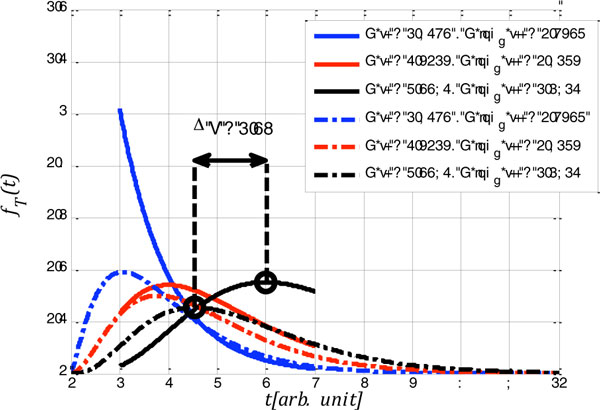


**Figure 2:**
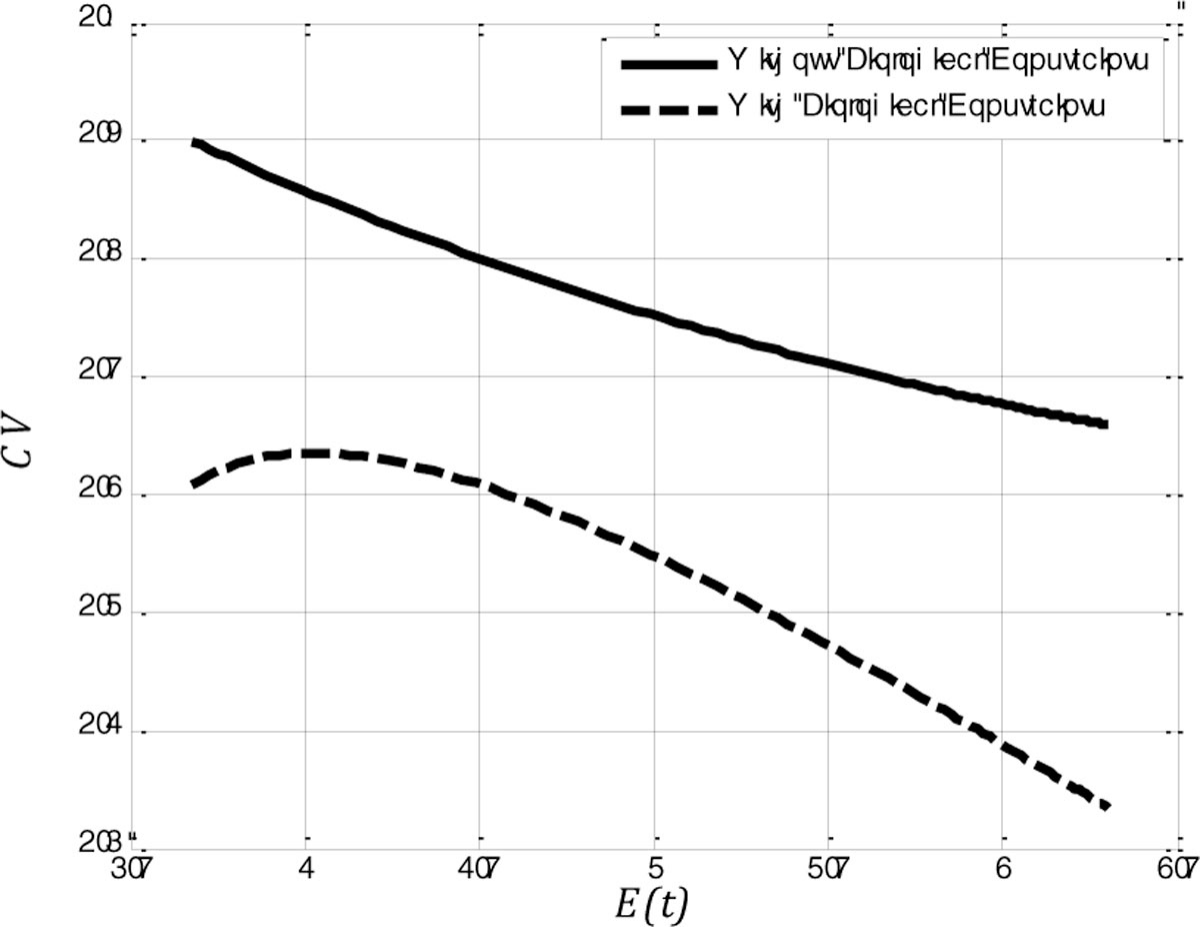


## References

[B1] Berger T Levy WB A Mathematical Theory of Energy Efficient Neural Computation and Communication IEEE Trans on Information Theory 2010 56 2 852 874

[B2] Xing J Berger T Sejnowski TJ A Berger-Levy energy efficient neuron model with unequal synaptic weights Proc of IEEE Int Symp on Information Theory 2012 2964 2968

